# Unveiling IoT Customer Behaviour: Segmentation and Insights for Enhanced IoT-CRM Strategies: A Real Case Study

**DOI:** 10.3390/s24041050

**Published:** 2024-02-06

**Authors:** Elaheh Eslami, Nazila Razi, Mahshid Lonbani, Javad Rezazadeh

**Affiliations:** 1 IT Department, Azad University, North Tehran Branch, Tehran 1667914161, Iran; elaheh.es64@yahoo.com; 2 School of Business and IT, Crown Institute of Higher Education, Sydney, NSW 2060, Australia; nazila.razi@cihe.edu.au; 3 IT Department, Kent Institute Australia, Sydney, NSW 2000, Australia; mahshid.lonbani@kent.edu.au

**Keywords:** Internet of things (IoT), data mining, customer preferences, customer satisfaction, customer segmentation, self-organizing map, decision tree

## Abstract

In today’s competitive landscape, achieving customer-centricity is paramount for the sustainable growth and success of organisations. This research is dedicated to understanding customer preferences in the context of the Internet of things (IoT) and employs a two-part modeling approach tailored to this digital era. In the first phase, we leverage the power of the self-organizing map (SOM) algorithm to segment IoT customers based on their connected device usage patterns. This segmentation approach reveals three distinct customer clusters, with the second cluster demonstrating the highest propensity for IoT device adoption and usage. In the second phase, we introduce a robust decision tree methodology designed to prioritize various factors influencing customer satisfaction in the IoT ecosystem. We employ the classification and regression tree (CART) technique to analyze 17 key questions that assess the significance of factors impacting IoT device purchase decisions. By aligning these factors with the identified IoT customer clusters, we gain profound insights into customer behaviour and preferences in the rapidly evolving world of connected devices. This comprehensive analysis delves into the factors contributing to customer retention in the IoT space, with a strong emphasis on crafting logical marketing strategies, enhancing customer satisfaction, and fostering customer loyalty in the digital realm. Our research methodology involves surveys and questionnaires distributed to 207 IoT users, categorizing them into three distinct IoT customer groups. Leveraging analytical statistical methods, regression analysis, and IoT-specific tools and software, this study rigorously evaluates the factors influencing IoT device purchases. Importantly, this approach not only effectively clusters the IoT customer relationship management (IoT-CRM) dataset but also provides valuable visualisations that are essential for understanding the complex dynamics of the IoT customer landscape. Our findings underscore the critical role of logical marketing strategies, customer satisfaction, and customer loyalty in enhancing customer retention in the IoT era. This research offers a significant contribution to businesses seeking to optimize their IoT-CRM strategies and capitalize on the opportunities presented by the IoT ecosystem.

## 1. Introduction

IoT technologies can enhance customer-centricity by providing real-time data and insights that enable businesses to better understand and cater to customer preferences. The use of advanced analytics can help extract meaningful information from IoT data to improve customer satisfaction, loyalty, and retention [1].

In the realm of IoT, everyday products, such as shampoo, are not exempt from the transformative potential of connected technologies. Shampoo holds a pivotal role in the daily routines of individuals worldwide. As an essential hygiene product for hair care, it enjoys universal consumption, positioning itself as a quintessential mass-market commodity [2]. IoT can revolutionize the way shampoo manufacturers approach their product. By incorporating IoT sensors and connectivity into shampoo bottles or packages, producers can gain real-time insights into customer usage patterns. These sensors could track factors such as frequency of use, preferred usage times, and even the specific hair types and needs of individual customers; with this wealth of data, companies can not only better understand and cater to customer preferences but also maximize profitability through more targeted product offerings. The responsibility for translating these IoT-driven devices into effective marketing campaigns and product innovations falls upon marketing experts. IoT technologies provide the data, but it is the marketing professionals who can harness this information to create personalized marketing strategies, offer tailored product recommendations, and enhance customer engagement [3].

The concept of customer segmentation, as introduced by Wendell R. Smith in 1956, has been a foundational approach in marketing and business. It offers a structured method for categorizing customers based on specific criteria, which is essential for tailoring marketing strategies and improving customer satisfaction [4]. Data mining is crucial across divers human activities, as it uncovers valuable, previously unknown patterns and knowledge (Gupta and Chandra, 2020) [5]. A self-organizing map (SOM) is like a special type of artificial neural network (ANN) that simplifies data into easy-to-understand maps. Unlike typical ANNs that fix mistakes, an SOM works by creating nodes to compete and rearrange information. It retains the original shape of the data, while making it simpler to spot patterns. It is handy for making complex data easier to visualize. SOM is indeed a popular technique used in data mining for clustering, visualisation, and dimensionality reduction tasks. SOMs can help identify patterns in customer behaviour by analyzing the data generated from IoT devices.

The SOM algorithm diverges from traditional biologically inspired neural network models by employing competitive learning, in which units vie for dominance, and by preserving topology through a neighborhood function that adjusts weights in parallel [6]. Over the years, various interactions of SOM models have found practical applications across diverse fields, ranging from meteorology and oceanography to finance, bioinformatics, and image retrieval [7]. In the IoT, these techniques can be applied to extract valuable insights from a wide range of data sources, making IoT a transformative technology in various domains.

In today’s intricate and competitive business landscape, systematic customer segmentation based on specific criteria enhances customer loyalty and broadens the portfolio of profitable customers, ultimately fostering long-term relationships [8]. Given the colossal volumes of customer data, data mining emerges as potent tool for evaluating comprehensive customer behaviour, particularly, in CRM applications [9]. In the context of data mining, clustering methods group data points, such that those within a cluster exhibiting greater similarity compared to those in other clusters, typically measured in terms of distance [10].

Furthermore, artificial intelligence (AI) techniques, including SOM, bee colony, particle swarm optimization (PSO), and genetic algorithm (GA) methods, have demonstrated the capability to effectively segment customers. While GA, rooted in evolutionary computing, harnesses processes such as mutation and crossing, its drawbacks lie in its time-consuming nature and the challenges of converging towards optimal solutions. On the other hand, SOM, a neural network-based unsupervised algorithm, relies on a trial-and-error approach but suffers from extended training times [11].

The utilisation of IoT technologies in the shampoo industry presents a transformative opportunity for business to revolutionize their understanding of consumer behaviour and preferences. However, the integration of IoT data into effective marketing strategies and product innovations poses a challenge, requiring the exploration and development of approaches to harness this data effectively in order to enhance customer satisfaction, loyalty, and retention. Accordingly, we develop the research question as follows: how can businesses leverage IoT technologies in the shampoo industry to revolutionize their comprehension of consumer behaviour and preferences, while effectively integrating IoT-derived data into marketing strategies and product innovations to enhance customer satisfaction, loyalty, and retention?

The overall aim of this study is to leverage IoT technologies, specifically IoT sensors incorporated into shampoo products, to collect real-time data on customer usage patterns. In doing so, the research seeks to effectively utilise Internet of things (IoT) technologies by embedding sensors into shampoo products. These sensors, integrated into the packaging or the product itself, are designed to continuously monitor and capture real-time data regarding how customers use the shampoo. The focus of the data collection is on customer usage patterns, encompassing factors such as the frequency of use, preferred times of use, and personalized preferences. The objective is to gain immediate and up-to-date insights into customer behaviour within the shampoo industry. By actively gathering information on usage patterns, the study aims to contribute to the understanding of consumer preferences, ultimately informing marketing strategies, product innovations, and enhancing overall customer satisfaction. This integration of IoT sensors into everyday products reflects a novel approach to navigating the dynamic and technologically driven market landscape, emphasizing the importance of real-time data in shaping business strategies. Therefore, to unravel these inquiries, we employ cluster analysis, identifying intriguing patterns that can inform marketing strategies for companies in the hair care product market. Our research entails a comprehensive analysis of customer preferences across several dimensions and involves a two-phase modeling approach. In the initial phase, we employ an SOM algorithm for segmentation, followed by the utilisation of the decision tree methodology in the second phase. Subsequently, we investigate customer behaviour by ranking factors affecting preferences within the cluster. This segmentation is critical for understanding customers based on their purchasing behaviour. Our study is dedicated to segmenting customers based on their purchasing behaviour and subsequently conducting experimental analysis using an SOM to infer insights into customer loyalty and customer return.

The scientific novelty of this paper lies in its pioneering approach to integrating IoT technologies into the shampoo industry, utilising IoT-enabled data collection to comprehensively understand and address consumer behaviour and preferences. By incorporating IoT sensors into shampoo products, this study revolutionizes conventional market analysis, allowing for the real-time tracking of usage pattens, such as frequency, preferred times, and personalized needs. It introduces an innovative paradigm in which IoT data, particularly analysed through the self-organizing map (SOM) algorithm, creates an opportunity to segment customers dynamically and precisely, transforming conventional marketing strategies into more tailored and personalized approaches. This intersection of IoT technologies and customer-centric marketing strategies within the shampoo market represents a groundbreaking advancement in leveraging data-driven insights for enhanced customer satisfaction and business competitiveness.

The remainder of this paper is structured as follows: Section 2 delves into a literature review on customer segmentation methods, while Section 3 provides detailed insights into the dataset. Section 4 and Section 5 includes information regarding data processing, clustering, experiments, and results. Finally, Section 6 offers a discussion of our findings and outlines future research avenues.

## 2. Literature Review

Customer segmentation is a widely explored area of market research (Das and Nayak, 2022) [10], typically focusing on categorizing customers based on their preferences or specific requirements. However, this study takes a novel approach by utilising the concept of customer lifetime value (CLV), offering a more efficient and practical means of segmentation. The research framework developed in this study involves segmenting customers, calculating the CLV for each segment, and estimating the future value (Nguyen et al., 2020) [12]. To achieve this, a two-tier clustering method is employed, leveraging a substantial database of customer transactions.

In a related study conducted by Zadeh et al., (2011) [13], the authors aimed to segment bank customers based on their behaviours to facilitate the bank’s retention strategies and acquisition efforts. The study utilised a comprehensive database comprising three interconnected tables. The first table contained demographic information, including age, gender, marital status, and other relevant attributes. The second table comprised transaction data, detailing customer transactions, while the third table provided information on bank issued cards. The study combined these attributes with transaction-specific details such as transaction type, frequency, service type, bank affiliation, and channel used (e.g., ATM, web, and terminal). Notably, Zadeh et al., (2011) categorized these factors based on their profitability, employing SOM to simplify complex high dimensional data. It is worth noting that the SOM approach involves a tradeoff between data presentation accuracy and topology preservation quality.

The concept of a decision tree, a structure resembling a traditional tree with root nodes, branches, and leaf nodes, is another relevant aspect discussed in the literature. Decision trees are used for attributes, testing at internal nodes, branching based on test outcomes, and labeling leaf nodes with class results. Common decision tree algorithms, such as ID3, CART, and C4.5, are frequently employed in data mining to create homogeneous nodes.

Classification and regression trees (CART) were introduced by Breiman et al., (1984), which were particularly distinctive in their construction of binary trees, with each internal node having precisely two outgoing edges. Splits are determined using the towing criteria, and the resulting tree is pruned using cost-complexity pruning. The CART method can also incorporate misclassification cost information and prior profitability distributions. Notably, it excels in generating regression trees, which predict real numbers rather than classes, minimizing squared prediction errors.

Some recent research, such as that by Mach-Krol and Hadasik (2021) [14] and Hassouna et al., (2016) [15], sought to compare data mining methods, specifically decision trees and logistic regression, in constructing a customer churn model. The study involved a real-world dataset from an English mobile operator, employing two balanced datasets, one for training, with 17 variables and 19,919 customers, and another for testing, with 17 variables and 15,519 customers. The authors utilised various decision tree algorithms, including CART, C5.0, and CHAID, for comparison with logistic regression. Evaluation criteria included the AUC of the ROC curve, top decile performance, and overall accuracy. Surprisingly, the C5.0 decision tree outperformed the other algorithms, including logistic regression, with an AUC of 0.763, contradicting earlier findings [16].

Customer segmentation, a critical marketing strategy, aims to group customers based on their shared behaviours, needs, and expectations. This allows businesses to gain deeper insights into their customers and tailor marketing strategies accordingly, maintaining more effective customer relationship management [12]. In this study, we will develop a predictive model capable of forecasting future customer sales using the proposed segmentation models. Competitive learning, a clustering technique, is among the methods employed to describe customer behaviour. Competitive learning identifies artificial representatives closely resembling objects within a specific cluster, with the Kohonen SOM being a notable application of this approach, as initially introduced by Kohonen (1982) [17].

The SOM algorithm, as a type of artificial neural network (ANN), stands out for its ability to simplify complex data, making it valuable for clustering, visualisation, and understanding patterns in customer behaviour generated from IoT devices. By elaborating on the significance of SOM in data mining for customer segmentation [18], our research aligns with the foundational concept of customer segmentation and underscores the evolution of this concept through the integration of advanced analytics, such as SOM, in the context of IoT [19].

According to Ref. [20], a novel approach is developed by incorporating the concept of customer lifetime value (CLV) for segmentation, offering an efficient means to categorize customers based on their future value. This approach builds upon existing research frameworks involving CLV and customer segmentation, providing a unique contribution to the literature. The paper [21] introduces a novel machine learning-based approach for sustainability assessment, utilising self-organizing map (SOM) clustering and classification and regression trees (CART). The study demonstrates high prediction accuracy in CART models and emphasizes the effectiveness of ensemble techniques. Evaluated on a sustainability assessment by fuzzy evaluation (SAFE) dataset, the proposed method showcases its potential as an automated decision-making tool for performance assessment, offering valuable insights into the integration of machine learning in addressing big data challenges [21].

In the realm of IoT customer behaviour, our research delves into the transformative potential of connected technologies, particularly within the context of customer relationship management (CRM) strategies. By leveraging IoT data and advanced analytics techniques, like SOM and classification and regression trees (CART), we scrutinize the intricacies of customer interactions with the connected devices. This analysis not only captures the dynamic patterns in IoT customer behaviour but also informs the development of tailored IoT-CRM strategies. Understanding the nuanced preferences and usage patterns revealed by IoT data empowers businesses to formulate personalized marketing approaches, offer targeted product recommendations, and enhance overall customer engagement. Thus, our research contributes to the evolving landscape of IoT-CRM strategies, providing insights that are crucial for businesses aiming to optimize their customer-centric initiatives and capitalize on the vast opportunities presented by the IoT ecosystem. By intertwining IoT customer behaviour analysis with strategic CRM considerations, our study stands as a valuable resource for organisations seeking to navigate and excel in the rapidly evolving digital marketplace.

## 3. Database Exploration: Unveiling Consumer Behaviour in the Shampoo Market

Within the dynamic hair care industry in Tehran, Iran, an in-depth investigation into consumer behaviour during shampoo purchases was conducted. Data collection was achieved by employing an online survey, meticulously crafted for this purpose. Administered through Google Docs, the survey yielded 207 valid responses. The questionnaire was structured into two parts. The first segment focused on collecting demographic information such as gender, marital status, age, family size, employment status, education level, and disposable income. The second segment delved into the myriad factors influencing shampoo purchases, consisting of 17 essential attributes, as depicted in Table 1. Respondents used a Likert scale (ranging from 1 to 5) to rate the importance of these attributes. The primary research goal in this phase was to apply clustering techniques to unveil distinct dimensions of customer preferences and priorities in the context of shampoo purchases.

The subsequent crucial step involved determining the appropriate number of clusters. Cluster indexing was achieved by utilising the mean of attributes within each cluster. Clustering processes were executed for two, three, and four clusters, and the results were validated by domain experts. The outcome identified three distinct clusters representing varying levels of consumer attitudes: very high importance, medium importance, and low importance. This segmentation became a valuable guide for shaping market strategies.

Objective Definition: Extracting Insights from Data Mining Techniques

Through framing research objectives by leveraging available data and data mining techniques, the study aimed to achieve the following:

(a) Customer Segmentation: Employing a self-constructed neural network (SOM) to cluster customers based on factors influencing their shampoo choices.
(b) Customer Classification: Utilising decision tree techniques to categorize customers based on factors influencing purchasing and product usage behaviour.
(c) Key Factor Identification: Pinpointing the most influential factors in shampoo purchases.
(d) Relative Prioritization: Determining the relative prioritization of these factors and analyzing their impact on consumer decisions.

This study offers a distinct advantage over traditional approaches, particularly in regards to customer segmentation. Incorporating the concept of customer lifetime value (CLV) enhances the segmentation process, making it more efficient and practical. Beyond customer segmentation, the research framework involves calculating CLV for each segment, providing a comprehensive understanding of customer dynamics. These methods offer increased precision, empowering companies to formulate effective strategies and cultivate a distinct vision for success in the industry.

## 4. Data Preprocessing and Clustering Analysis

In the data preprocessing phase, careful curation of the dataset involved selecting 17 relevant items out of the initial 22. Systematic removal of null values and exclusion of travel records with a duration of 0 between January 2017 and 2018 refined the dataset to 207 data points, with 17 essential attributes. These data points formed the basis for subsequent clustering analyses, resulting in the distribution of participants in each cluster: Cluster 1 (42), Cluster 2 (106), and Cluster 3 (59).

### 4.1. Robustness of SOM and CART Techniques

The application of self-organizing maps (SOM), an unsupervised learning algorithm, and classification and regression trees (CART) was integral to the clustering analysis. SOM excels in organizing complex, high-dimensional data into visually understandable maps, showcasing adaptability across diverse fields. Conversely, CART efficiently generates regression trees, reducing prediction errors and proving valuable for decision-making scenarios. Statistical validation and comparative analysis played a crucial role in substantiating the effectiveness of these techniques.

Comparative Analysis: Beyond SOM and CART, exploration of alternative methods, such as k-means clustering and deep learning networks, was imperative. Evaluation based on performance metrics provided a comprehensive understanding of their applicability.

Performance Metrics: Defining a comprehensive set of performance metrics, including accuracy, precision, recall, F1 score, and computational time, validated the chosen techniques. Comparative analysis across different methods helped identify the most suitable approach for segmenting IoT-driven customer behaviour.

This research aims to provide a robust understanding of customer behaviour within the IoT-enabled shampoo market by highlighting strengths and practical applications and substantiating effectiveness through statistical validation and comparative analyses.

#### 4.1.1. Utilising Self-Organizing Maps (SOM) for Customer Clustering

In this section, SOM, a neural network technique, was employed to cluster customers based on their shampoo preferences. The analysis considered three clusters representing customers exhibiting preferences with very high, average, or low importance across the studied attributes.

Characteristics and Behaviours of Customer Clusters: Beyond their inclination for IoT device adoption, the three customer clusters exhibit distinct characteristics and behaviours relevant to shampoo preferences:

(a) Cluster 1 (high importance): Prioritizes specific attributes such as cleansing efficacy, consistent quality, possessing herbal properties, and brand familiarity. This cluster emphasizes product quality, brand recognition, and specific functional attributes in their shampoo choices.
(b) Cluster 2 (medium importance): Assigns moderate importance across most attributes, seeking a balanced mix without extreme emphasis on any specific factor.
(c) Cluster 3 (low importance): Assigns lower importance to most attributes, focusing more on basic functionality and pricing.

#### 4.1.2. Deeper Analysis and Implications

Understanding these cluster-specific behaviours provides critical insights for market segmentation strategies and product development within the shampoo industry:

- Product Tailoring: Cluster 1 customers seek high-quality, branded products with specific functional attributes.
- Balanced Offerings: Cluster 2 customer preferences for a balanced mix suggests the need for versatile product offerings.
- Value-Based Approach: Cluster 3 customers’ focus on basic functionalities and pricing signals a value-based approach.

These clusters serve as a guide for formulating targeted marketing strategies and developing product lines aligning with different customer needs and preferences within the shampoo market.

The summary of the clustering results is provided below:

In the context of high-dimensional input spaces, SOM plays a crucial role in generating a lower-dimensional representation of the training data. ‘S’ indicates the set of training points residing in an n-dimensional input space, while the latent space is defined as (m − 1) dimensional. Within this latent space, we have ‘D’ neurons, represented by D = D_1_ × … × D_m−1_. Each of these neurons, labeled as ‘u’ and ranging from 1 to D, possesses two key attributes.

1. A predefined position in the latent space denoted as z*^u^* = ($z_{1}^{u}$,…, $z_{m-1}^{u}$), where $z_{i}^{u}$ ∈ {1, ⋯, D*_i_*} for *i* = 1, ⋯, *m* − 1.
2. A representation or weight vector, defined as w*^u^* = ($w_{1}^{u}$, …, $w_{n}^{u}$), which resides within the input space.

**Figure 1:**
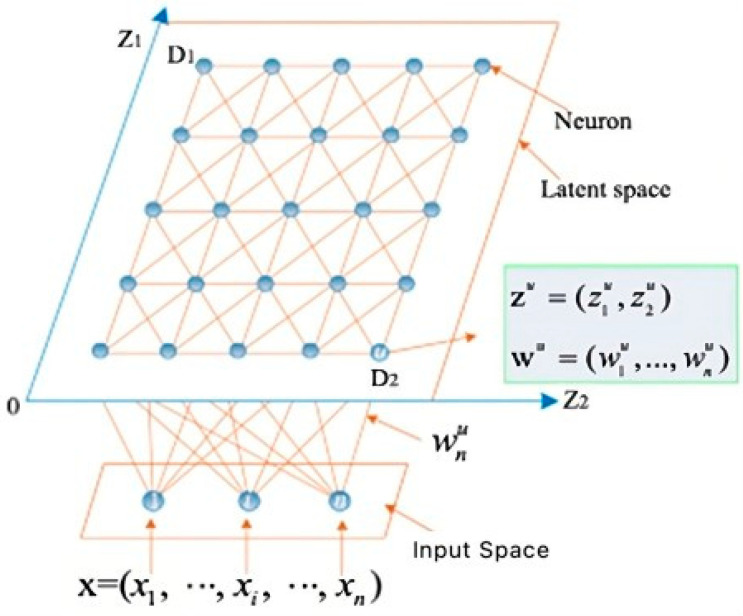
An illustration of a two-dimensional SOM.

Figure 1 illustrates a typical SOM topology. In this example, the input space is n-dimensional, while the latent space is two-dimensional [22]. The primary objective of SOM is to identify the representation vectors that are embedded within the latent space through the training data. 

The SOM learning process is initiated with the assignment of randomly selected training from the set ‘S’ to each neuron’s weight vector. These weight vectors are subsequently updated by iteratively considering training points that are in proximity to them [15].

### 4.2. Assessing Data Clustering Tendency

Before embarking on our experimentation and the application of any clustering technique to our database, it is essential to examine the inherent tendency of the data to form clusters or exhibit similarities among objects. These diverse tendencies must be carefully analysed, as they influence a company’s decision to select specific customer segments and customize their marketing strategies to align with unique customer needs.

**Figure 2:**
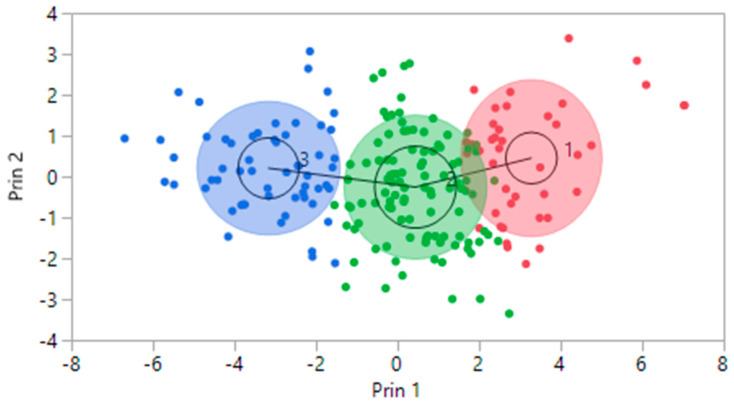
Clusters using the principal component analysis-based visualisation method.

Furthermore, we can visualize the clustering results in a comprehensive two-dimensional representation using dimensions derived from principal component analysis (PCA). In pursuit of this objective, we condensed the 17 purchasing stimulus characteristics utilised in the clustering process by applying PCA to instill essential information into two key dimensions (refer to Figure 2). Notably, the SOM method, employed in our analysis, possesses the capability to effectively distinguish and delineate clusters within the data, enhancing our understanding of the underlying patterns and groupings.

### 4.3. Self-Organized Maps (SOM)

Visualisation of high-dimensional databases is a complex task, often fraught with challenges due to the sheer number of features and data points involved. In this study, we employ SOM, also known as Kohonen maps, to simplify the presentation of high dimensional data and to explore meaningful relationships within it. The SOM, a form of artificial neural network, serves as a powerful tool for transforming multidimensional data into a visually intuitive two-dimensional representation, elucidating the intrinsic relationships among data objects.

The fundamental strategy underlying SOMs is to map data objects onto a two-dimensional lattice grid in such a way that their positions reflect their similarity in the original feature space. This not only simplifies the visualisation of complex data but also facilitates the clustering of similar data points. By transforming high-dimensional data into a map, we make visualisations more accessible and aesthetically pleasing.

At the core of the SOM method lies competitive learning. For each sample vector, weights of the same dimension as the output network (equal to the number of nodes) are initialized randomly. Subsequently, the Euclidean distance, a commonly used metric, is computed between the sample vector and these weights. The node with the minimum distance is declared the winner, effectively representing a cluster of similar objects, or a neighborhood. Following the competitive phase is the adaptation phase, in which the wights of all neighboring nodes are adjusted. Importantly, the learning rate decreases progressively as a function of training epochs, ensuring a gradual convergence towards the optimal representation.

**Figure 3:**
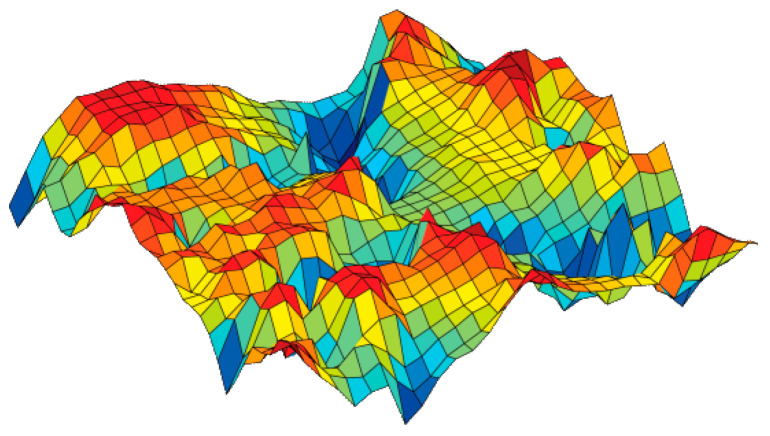
Visualisation of market basket data, according to Heskes.

Tom Heskes (2001) [23] demonstrated a direct correspondence between minimum distortion topographic map formation and maximum likelihood Gaussian mixture density modeling, particularly in the homoscedastic case. This research builds upon the traditional distortion (vector quantization) formulation of the self-organizing map. Heskes’ work showcases the practical applications of SOMs, including market basket analysis. In this context, the objective is to map products onto a two-dimensional lattice in such a way that proximate products exhibit similarities in regards to purchasing behaviour. Products with similar conditional probabilities of being purchased together are clustered together on the map. In another application, Heskes explores the use of SOMs in analyzing supermarket transactions. Here, products are grouped into categories, and co-occurrence frequencies are considered. The result is a two-dimensional density map that reveals clusters of products that are frequently purchased together, such as prominent clusters of household products (as illustrated in Figure 3). This demonstrates the versatility and power of SOMs in uncovering meaningful patterns within high-dimensional data, with implications for various fields, including market analysis and product recommendation systems.

**Figure 4:**
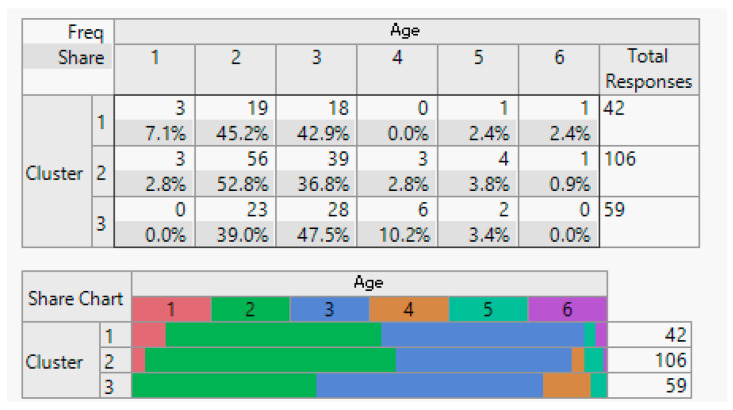
Gender demographic breakdown (male/female).

**Figure 5:**
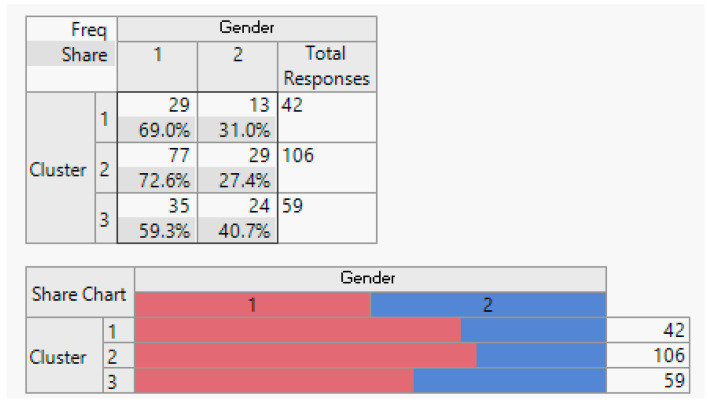
Age demographic range (20 to 40).

**Figure 6:**
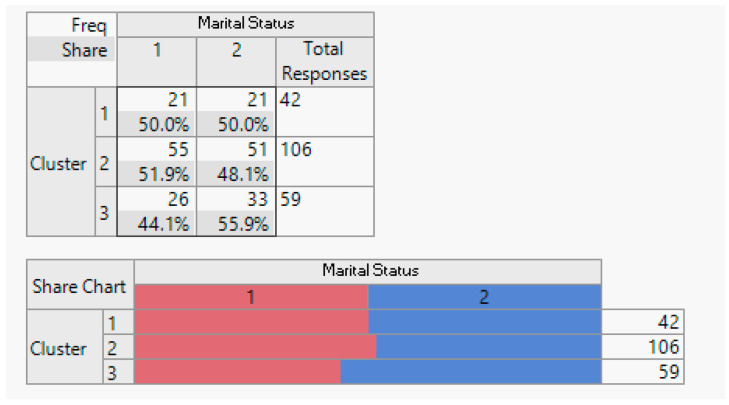
Marital status demographic breakdown (single/married).

**Figure 7:**
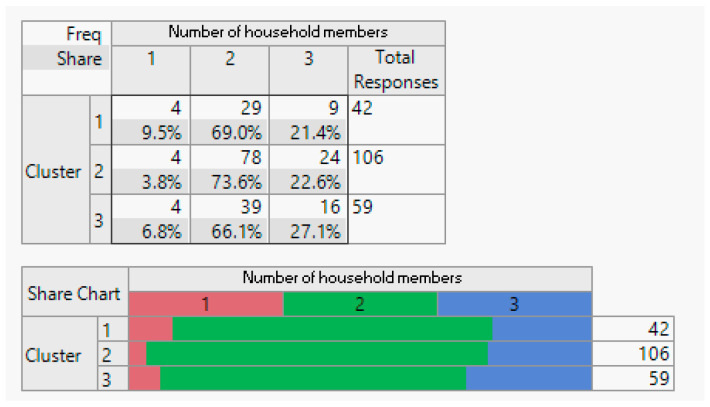
Household demographic data (number of household members).

**Figure 8:**
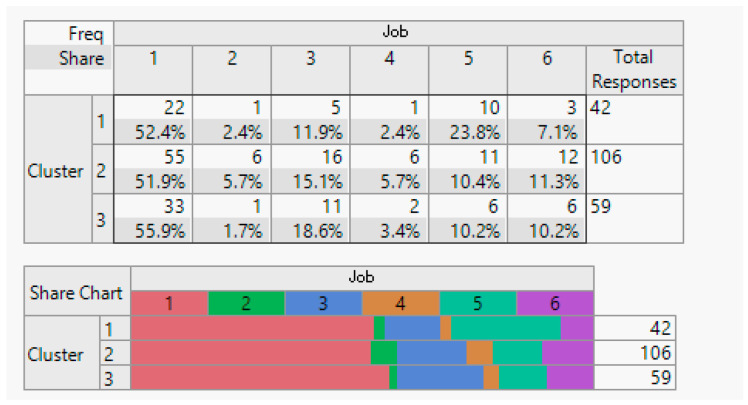
Employment status demographic breakdown (employed/unemployed).

**Figure 9:**
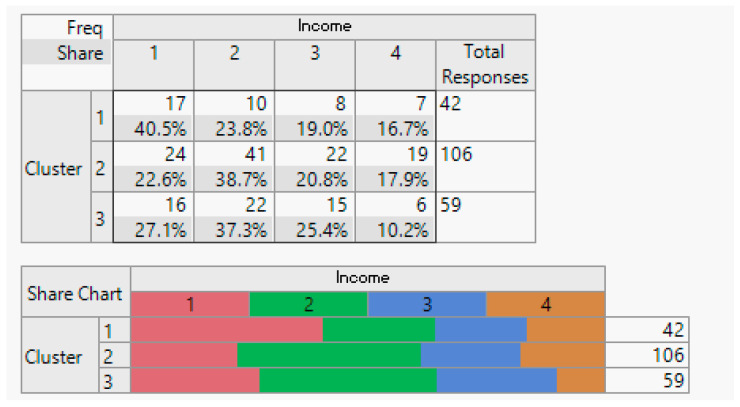
Income demographic segmentation.

**Figure 10:**
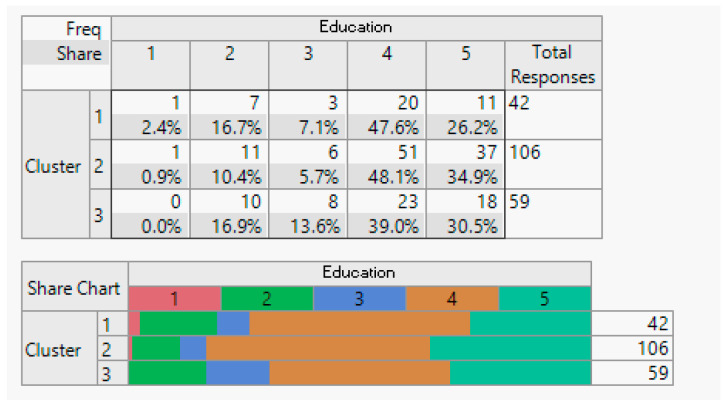
Overview of education demographics.

The color scheme in the above illustration transitions from red to dark blue, symbolizing the average Euclidean distance between adjacent nodes. The SOM grid, depicted in Figure 3, serves as a visual representation allowing for a comprehensive understanding of the dataset distribution. Within this visual depiction, red areas denote dissimilarity, indicating substantial Euclidean distances between objects, while dark blue areas signify similarity, denoting minimal Euclidean distances between objects. The colour gradient, progressing from orange to blue, visually communicates the reduction in distance between nodes. Moreover, this colour scheme offers the flexibility to visualize individual features or attributes. In this context, each colour corresponds to the value of a specific attribute, with dark blue representing low values and red representing high values. Figure 1, Figure 2, Figure 3, Figure 4, Figure 5, Figure 6, Figure 7, Figure 8, Figure 9 and Figure 10 provides a visual representation of assertions above. The approach that was discussed enhances the interpretability and insight into the dataset, providing a nuanced understanding of both overall distribution patterns and individual attribute variations.

### 4.4. Experiment 1: Clustering by SOM

Execution: Following the clustering methodology described earlier, we conducted clustering experiment with two, three, and four clusters. After consulting with domain experts, we determined that three clusters provided the most meaningful results. The outcomes of this clustering are presented below.

Evaluation: Table 1 displays the average importance of each question. Notably, customers in Cluster 1 consistently scored above 4 points across all studied features. In Cluster 2, the same customers scored above 3 points, and in Cluster 3, customers had an average score of less than 3 for all features.

Analysis: To facilitate the interpretation of the clustering results, we visualised the clusters, attributes, and the target class as a scatter plot for the top 17 informative attributes. The analysis results are depicted in the following figures.

### 4.5. Experiment 2: Decision Tree by CART

Classification: Classification is the process of categorizing newly encountered entities into predefined classes by analyzing their features. It involves making informed decisions based on past examples [24]. One popular classification technique is the use of a decision tree.

Decision trees: A decision tree is a tree-like structure in which each internal node represents a feature and a split point, while each leaf node corresponds to a class label. These trees are constructed from training data and are used to classify new instances [24]. Decision tree learning methods are widely applied in data mining, aiming to predict target variable values based on input attributes. The process begins by selecting an attribute that efficiently splits the data, which then becomes the root node. The attribute with the highest information gain is chosen as the splitting attribute [25].

CART Algorithm: The CART algorithm, developed by Quinlan [26], employs information gain as its splitting criterion. The topmost node in the tree is the root node, and the attribute with the highest information gain is selected as the split attribute. The tree is built from training instances and is used for classifying test data. When information gain becomes minimal, or when all instances belong to a single target class, tree growth halts [24]. CART introduces the concept of a cost function, given by Ref. [27]: *R*α(*T*) = *R*(*T*) + α, where *R*(*T*) represents the misclassification cost of the sub-tree after pruning a specific branch, and α is the cost complexity parameter. Gradually increasing α, starting from 0, leads to a series of nested trees decreasing in size. The optimal tree size is determined through 10-fold cross-validation, where the dataset is divided into subsets. One subset is used as an independent test set, while the others are used for training. The tree growing and pruning process is repeated N times, and the tree with the lowest misclassification error is considered optimal. Additionally, Breiman’s 1-SE rule is used to select the smallest tree within one standard error of the minimum misclassification error. CART is implemented in the R system as RPART [28].

Application and Insights: CART analysis serves as a valuable tool for researchers, allowing them to identify key variables for potential interventions and decision making. For instance, it reveals the significant role of the cleaning property, achieving a high prediction success rate of approximately 90%. Furthermore, the resulting model exhibits a high true negative rate, and meaningful rules are extracted from the dataset, showcasing the efficacy of the decision tree method in predicting customer preferences in the hair care industry.

Correlating Customer Importance and Classification: In this experiment, we aimed to establish a correlation between customer-related variables used in clustering and their importance class using the CART decision tree technique within SPSS software (version 26). The GINI index serves as the impurity criterion in the CART model, and tree pruning is applied. Based on the target variable, the decision tree is constructed. Since classification is a data-driven and supervised data mining approach, a target variable is essential. Building upon the results obtained from previous customer segmentation modeling, we utilise the groups derived from clustering based on customer behaviour and characteristics as categorical target variables. It is worth noting that the classification is performed separately for customers seeking excellent, medium, and low quality products [29]. The ultimate objective is to establish a connection between the variables used in clustering, describing a customer’s preferences, and their respective importance classes [30].

## 5. Results of Model Implementation

**Figure 11:**
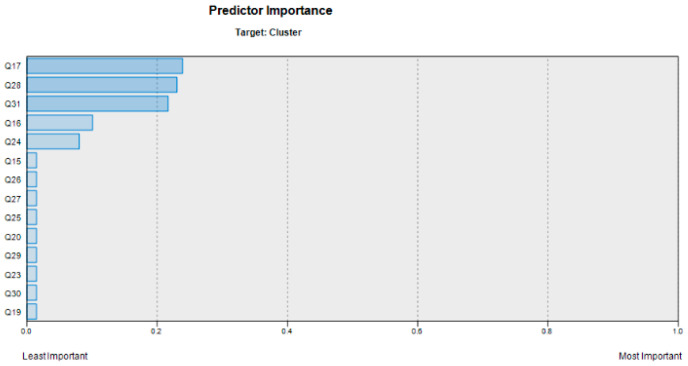
Overview of investigated factor prioritization.

**Figure 12:**
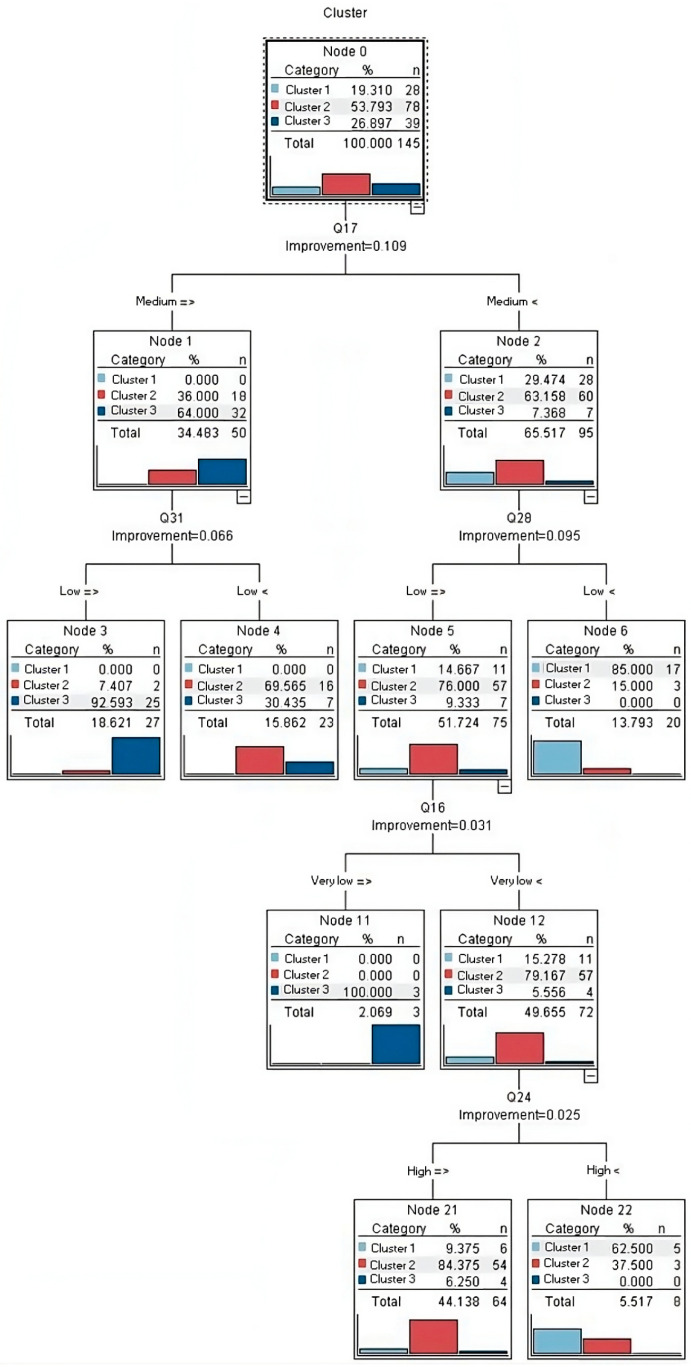
Decision tree model developed in the current study.

Upon applying the CART decision tree technique to classify the factors outlined in Table 2, the results revealed valuable insights into their impact on customer satisfaction. Figure 11 provides a visual representation of the factors ranked by their influence on customer satisfaction, while Figure 12 represents the decision tree, showcasing how each factor contributes to the model’s predictions.

As illustrated in Figure 12, the cleansing ability of the shampoo (question 17) emerges as the primary determinant in customer satisfaction. At the top level of the decision tree, it becomes evident that when customers express a satisfaction level of approximately 30% regarding the shampoo’s cleansing properties, they invariably belong to the high-satisfaction customer class. Furthermore, if satisfaction with this aspect surpasses 63%, irrespective of the status of other examined factors, customers are unequivocally placed in the category of average satisfaction. This finding underscores the pivotal role of shampoo cleansing efficacy in shaping overall customer satisfaction.

As depicted in Figure 12, the influence of various factors on customer satisfaction are as follows:

- First Priority: The significance of shampoo cleansing properties.
- Second Priority: The relevance of lotto draws, prizes, and discounts.
- Third Priority: The importance of product diversity.
- Fourth Priority: The value of moisturizing properties.
- Fifth Priority: The significance of shampoo volume.

While Figure 12 presents additional factors within this context, our primary focus and interpretation lie with the above priorities. These findings provide the shampoo company with valuable insights for enhancing customer satisfaction. By recognizing the relative importance and prioritization of factors influencing customer satisfaction, the company can strategically allocate resources and efforts to prioritize the most impactful and higher-priority factors, thereby optimizing results efficiently and cost-effectively.

In addition to items discussed in the previous sections, it is important to consider the fact that emerging technological trends to have the potential to influence customer preferences and behaviour in the following ways: firstly, the advent of new IoT devices and applications constantly introduces novel ways for individuals to interact with technology. This interaction, in turn, impacts the expectations and preferences of customers. For instance, the integration of AI-driven personalization in smart devices or the increase in edge computing can significantly alter user experiences, influencing their choices and satisfaction levels. Secondly, IoT technologies are rapidly changing; however, the ultimate aim is determining how they can increase the required performance. Whenever new data becomes available, these techniques can be used to perform a new analysis or evaluation. As technological trends evolve, the ability to rapidly integrate new techniques into existing data analysis processes becomes a strategic advantage. This adaptability not only refines models but ensures that companies remain at the forefront of industry innovations, contributing to a more robust and future-proof success trajectory. Achieving higher accuracy for these models will help companies plan better sales strategies, enhance customer satisfaction, and develop a clearer vision for success in the industry.

## 6. Discussion and Conclusions

In this extensive study, our focus has been on gaining a profound understanding of the preferences and priorities of shampoo buyers, leading to pivotal insights that intersect significantly with the transformative potential of IoT technology. The insights derived from our investigation into customer preferences within the shampoo market underscore the substantial impact IoT technology can have on the industry. Through the utilisation of real-time data, enhanced segmentation techniques, and the delivery of valuable insights, IoT emerges as a catalyst for revolutionizing strategies. This empowerment allows companies to optimize their approaches and thrive in the competitive landscape of the shampoo industry, marking the onset of a new era characterized by informed decision-making and customer-centric strategies. Our study identified specific areas where the synergy of consumer insights and IoT potential can reshape the industry:

(a) Data Collection and Analysis: The extensive database compiled in this study, encompassing the demographic and behavioural attributes of shampoo buyers, lays the foundation for a paradigm shift. Further integration with IoT-enabled shampoo products could revolutionize data collection by extending research methods beyond the use of traditional demographics to capture real-time usage patterns. IoT devices, by tracking how and when customers use shampoo, along with their product preferences, have the potential to enrich analysis, providing dynamic insights into consumer behaviour.
(b) Customer Clustering: The segmentation of customers into distinct clusters based on preferences emerges as a valuable tool. In an IoT landscape, this approach can evolve to become more dynamic and precise. Continuous data collection through IoT devices enables the real-time identification of changing consumer preferences, empowering companies to adapt swiftly and tailor products accordingly.
(c) Quality and Product Attributes: Emphasizing product quality and attributes such as cleansing properties remains pivotal in addressing customer preferences. In an IoT context, smart shampoo packaging could gather and transmit usage data, enabling companies to monitor and maintain product quality. IoT technology ensures that products consistently meet customer expectations, a pivotal factor for sustained satisfaction and loyalty.
(d) Decision Tree Insights: The decision tree analysis underscored the crucial role of specific shampoo attributes in customer segmentation. IoT integration could further enhance this by providing real-time data regarding customer satisfaction and preferences, empowering targeted marketing efforts to bolster satisfaction and loyalty.
(e) Competitive Advantage: IoT-enabled understanding of customer preferences offers companies a competitive edge. Leveraging IoT for data gathering and analysis enables tailored strategies that enhance customer satisfaction, paving the way for long-term success in the competitive shampoo industry. Personalized marketing, refined product development, and improved customer retention become achievable using this approach.

This study’s achievements lay a solid groundwork for a paradigm shift in the shampoo industry, showcasing the potential of IoT integration in shaping consumer-centric strategies. The proposed approach not only offers immediate advantages in terms of competitive positioning but also suggests further research frontiers, promising ongoing evolution and innovation within this domain. By integrating consumer insights with IoT potential, this transformative paradigm offers a competitive edge, allowing companies to tailor strategies, optimize product development, and fortify customer relationships in a dynamically evolving market. Understanding customer preferences through IoT data has the potential to offer avenues for personalized marketing, enhancing customer satisfaction and fostering long-term loyalty. Another notable benefit is the continuous monitoring of product quality through IoT integration, ensuring alignment with customer expectations, a pivotal factor for sustained customer satisfaction.

The exploration conducted in this study does not culminate here, but rather it opens doors for expansive future research. Further studies could delve into the intricacies of integrating IoT into shampoo products for robust data collection, examining technical feasibility and consumer privacy concerns. Research focusing on real-time adaptation strategies based on continuously evolving consumer preferences in an IoT context could redefine market responsiveness. Investigating how IoT-driven insights influence long-term consumer behaviour and brand loyalty can offer deeper insights into sustained market success, paving the way for continued innovation and evolution in the shampoo industry.

In the quest to enhance business strategies within the shampoo industry, practical recommendations emerge to guide businesses in the effective implementation of IoT-enabled customer-centric approaches. First and foremost, personalized product offerings stand out as a pivotal strategy. Through the utilisation of real-time data collected by IoT-enabled shampoo products, businesses can capture usage patterns and attribute preferences, allowing for tailored product formulations, packaging designs, and bundle offers based on individual customer needs. This discerning recommendation encourages investment in IoT devices to gather real-time insights into customer preferences, thereby enabling personalized product development. Additionally, dynamic marketing campaigns become a powerful tool when driven by IoT-generated consumer insights. Leveraging IoT data empowers businesses to create targeted campaigns that resonate with customer preferences. The ability to adapt marketing content and channels dynamically based on real-time feedback from IoT-connected products enhances the recommendation to employ IoT analytics. This, in turn, personalizes marketing efforts, fostering heightened customer engagement and brand loyalty. Moreover, enhancing the customer experience becomes a key focus by leveraging data-backed customization through IoT insights. By tailoring the customer experience based on individual preferences captured through IoT-connected devices, businesses can implement personalized recommendations, such as usage tips or complementary product suggestions. This perceptive recommendation advocates for the integration of IoT-generated insights into customer interactions, thereby enhancing overall satisfaction and fostering long-term loyalty.

On the product development front, embracing an agile approach through real-time quality monitoring becomes crucial. Deploying IoT sensors in product packaging enables companies to monitor attributes like freshness, quality, and effectiveness, gathering real-time data to iteratively enhance product formulations based on customer feedback. The recommendation urges the utilisation of IoT-driven quality monitoring to consistently improve product offerings, meeting evolving customer expectations. Data-driven customer service emerges as another strategic avenue. IoT devices can alert companies to usage issues or product deficiencies in real time, allowing for the establishment of responsive customer service protocols backed by IoT data. This insightful recommendation encourages businesses to employ IoT perceptions for proactive and personalized customer support, ultimately enhancing the overall customer experience and fostering loyalty. Expanding beyond customer-centric strategies, integrating IoT into business operations offers further advantages. Supply chain optimization, facilitated by real-time inventory management through IoT sensors, streamlines operations and ensures improved stock management. Strategic partnerships with IoT technology providers or data analytics firms can enhance data interpretation and drive better business decisions, resulting in the recommendation to collaborate with IoT experts for optimal use of IoT-generated data. Lastly, prioritizing compliance and security in handling customer information collected through IoT devices is crucial. Implementing stringent security measures and adhering to data protection regulations are recommended to build consumer confidence in IoT-enabled products.

These detailed strategies collectively aim to guide businesses in effectively leveraging IoT technology within the shampoo industry. Embracing IoT-driven insights not only enriches customer experiences but also optimizes business operations, fostering growth and a competitive advantage in a dynamic market landscape. Moreover, In essence, the transformative paradigm we propose, involving the integration of consumer insights with IoT potential, is a versatile approach that can guide businesses in various digital domains. The principles outlined in our study not only offer immediate advantages in terms of competitive positioning but also pave the way for ongoing evolution and innovation in the digital landscape. Future research in different industries can build upon these principles, exploring the intricacies of IoT integration and its impact on customer-centric strategies and business operations.

## Figures and Tables

**Table 1:** Consumer attributes influencing shampoo purchases.

Question	Attributes
1	Fragrance
2	Moisturizing effectiveness
3	Cleansing efficacy
4	Anti-dandruff properties
5	Hypoallergenic
6	Transparency
7	Herbal trait
8	Price
9	Brand familiarity
10	Volume of content
11	Advertising
12	Family/friend recommendations
13	Seller advice and suggestions
14	Lotto draw, prize, and discount
15	Shampoo container design
16	Consistent in quality
17	Product variety

**Table 2:** Consumer attribute cluster analysis.

Attributes	Cluster 1 (High Importance) C-SOM-1-1	Cluster 2 (Medium Importance) C-SOM-1-2	Cluster 3 (Poor Importance) C-SOM-1-3
Number of Records	42	106	59
Fragrance	3.55	3.22	2.27
Moisturizing effectiveness	3.98	3.28	2.22
Cleansing efficacy	4.62	4.02	2.81
Anti-dandruff properties	4.05	3.01	2.15
Antiallergy	4.21	3.54	2.02
Transparency	4.14	3.55	2.34
Herbal traits	4.31	3.30	2.29
Price	3.71	3.12	2.49
Brand familiarity	4.36	3.73	2.69
Volume of content	4.07	3.24	2.10
Advertising	3.60	2.64	1.54
Family/friend recommendation	3.71	3.07	2.03
Seller advice and suggestions	3.64	2.56	1.90
Lotto draw, prize, and discount	2.95	1.52	1.19
Shampoo container design	3.31	2.12	1.47
Consistency in quality	4.79	4.32	3.15
Product variety	3.95	3.19	1.86

## Data Availability

Data are contained within the article.
